# Evaluating handwriting characteristics as a novel assisted screening approach for Mild Cognitive Impairment in community settings

**DOI:** 10.1002/alz.092249

**Published:** 2025-01-09

**Authors:** Lulu Shi, Yinxia Ren, Zhuqin Wei, Hengnian Qi, Ruoyu Zhang, Lina Wang

**Affiliations:** ^1^ Huzhou University, Huzhou, Zhejiang China

## Abstract

**Background:**

Older adults with Mild Cognitive Impairment (MCI) show decreased flexibility, speed, and fluency in writing, suggesting the feasibility of using quantitative handwriting analysis for MCI detection. Given task variability and the complexities of Chinese versus English characters, investigating Chinese handwriting characteristics is crucial for refining MCI screening approach.

**Method:**

259 participants (109 with MCI, 150 controls) were engaged to completed six handwriting tasks using a dot‐matrix digital pen, encompassing four Chinese characters tasks and two graphical drawing tasks. Kinematic handwriting parameters were collected and analyzed using Light GBM, XGBoost, RF, and SVM machine learning models to assess their effectiveness in MCI recognition.

**Result:**

A total of 48 handwriting characteristics were extracted, of which 12 characteristics derived from graphical drawing tasks and 18 from Chinese characters writing tasks exhibited significant differences between the two groups (P<0.05). Compared to the control group, the MCI group demonstrated a significantly longer mean lag time (Z = ‐2.991, P = 0.003 for graphical drawing tasks; Z = ‐6.441, P<0.001 for Chinese characters tasks), lower average writing velocity for graphical drawing tasks (Z = ‐3.263, P = 0.001), shorter single stroke lengths (Z = ‐2.352, P = 0.019), and decreased writing correctness scores (Z = ‐9.388, P<0.001 for graphical drawing tasks; Z = ‐5.949, P<0.001 for Chinese characters tasks). The accuracy of XGBoost, **Light GBM**, RF, and SVM machine learning models constructed based on handwriting characteristics were 81.35%, **83.28%**, 81.03%, and 63.02%, respectively, with their Area Under the Curve (AUC) values being 0.888, **0.896**, 0.895, and 0.570.

**Conclusion:**

Handwriting characteristics in Chinese writing tasks showed promise for MCI screening in older adults, with the Light GBM model offering improved predictive accuracy, potentially enhancing MCI detection among community‐dwelling older adults.

**Trial registration**: The trial was registered at Chinese Clinical Trials Registry on 05/02/2022 (www.chictr.org.cn; Number Registry: ChiCTR2200059499).

**Funding**: National Natural Science Foundation of China (NO.72174061; NO.71704053), China Scholarship Council Foundation (NO. 202308330251).

